# Antihyperglycemic Properties of Extracts and Isolated Compounds from Australian *Acacia saligna* on 3T3-L1 Adipocytes

**DOI:** 10.3390/molecules28104054

**Published:** 2023-05-12

**Authors:** Anjar P. Asmara, Anchalee Prasansuklab, Anchalee Chiabchalard, Hui Chen, Alison T. Ung

**Affiliations:** 1 School of Mathematical and Physical Sciences, Faculty of Science, University of Technology Sydney, Ultimo, NSW 2007, Australia; anjarpurba.asmara@student.uts.edu.au; 2 Natural Products for Neuroprotection and Anti-Ageing Research Unit, Chulalongkorn University, Bangkok 10330, Thailand; anchalee.pr@chula.ac.th (A.P.); anchalee.c@chula.ac.th (A.C.); 3 College of Public Health Sciences, Chulalongkorn University, Bangkok 10330, Thailand; 4 Department of Clinical Chemistry, Faculty of Allied Health Sciences, Chulalongkorn University, Bangkok 10330, Thailand; 5 School of Life Sciences, Faculty of Science, University of Technology Sydney, Ultimo, NSW 2007, Australia; hui.chen-1@uts.edu.au

**Keywords:** *Acacia saligna*, (−)-epicatechin, naringenin-7-O-α-*L*-arabinopyranoside, 3T3-L1 adipocytes, ROS, mt-ROS, mitochondrial membrane potential, glucose uptake, AMPK-α, GLUT-4

## Abstract

Our early work indicated that methanolic extracts from the flowers, leaves, bark, and isolated compounds of *Acacia saligna* exhibited significant antioxidant activities in vitro. The overproduction of reactive oxygen species (ROS) in the mitochondria (mt-ROS) interfered with glucose uptake, metabolism, and its AMPK-dependent pathway, contributing to hyperglycemia and diabetes. This study aimed to screen the ability of these extracts and isolated compounds to attenuate the production of ROS and maintain mitochondrial function via the restoration of mitochondrial membrane potential (MMP) in 3T3-L1 adipocytes. Downstream effects were investigated via an immunoblot analysis of the AMPK signalling pathway and glucose uptake assays. All methanolic extracts effectively reduced cellular ROS and mt-ROS levels, restored the MMP, activated AMPK-α, and enhanced cellular glucose uptake. At 10 µM, (−)-epicatechin-**6** (from methanolic leaf and bark extracts) markedly reduced ROS and mt-ROS levels by almost 30% and 50%, respectively, with an MMP potential ratio 2.2-fold higher compared to the vehicle control. (−)-Epicatechin **6** increased the phosphorylation of AMPK-α by 43%, with an 88% higher glucose uptake than the control. Other isolated compounds include naringenin **1**, naringenin-7-O-α-*L*-arabinopyranoside **2**, isosalipurposide **3**, *D*-(+)-pinitol **5a**, and (−)-pinitol **5b**, which also performed relatively well across all assays. Australian *A. saligna* active extracts and compounds can reduce ROS oxidative stress, improve mitochondrial function, and enhance glucose uptake through AMPK-α activation in adipocytes, supporting its potential antidiabetic application.

## 1. Introduction

*Acacia saligna* (Labill.) H. L. Wendl. is a shrub or small tree (2–10 m) with grey to red-brown bark, linear to lanceolate (8–25 × 0.4–2 cm) and green to glaucous leaves, and round, bright yellow flowers (5–10 mm diameter). It usually flowers between August and October and produces mature legumes from November to January. It is native to Western Australia and was previously named *A. cyanophylla* Lindl, *A. bracteate* Maiden & Blakeley, *A. lindleyi* Meissner, *Mimosa saligna* Labill., and *Racosperma salignum* (Labill.) Pedley [1].

Our previous work on the bioactive extracts of Australian *A. saligna* revealed that methanolic extracts and isolated compounds have significant antioxidant effects and can inhibit the yeast α-glucosidase. The results indicate the potential of this plant as a rich source of bioactive compounds, such as flavonoid and cyclitol groups, to target type 2 diabetes [2].

In type 2 diabetes, insulin resistance interrupts glucose homeostasis, leading to hyperglycaemia. It is characterised by disrupted glucose uptake in insulin-sensitive cells due to impaired glucose transporter 4 (GLUT-4) expression and translocation to the cell membrane. The overproduction of hyperglycemia-induced cellular reactive oxygen species (ROS) correlates with altered GLUT-4 trafficking in 3T3-L1 adipocytes [3]. This study suggested that ROS can directly oxidise thiol (SH) groups (cysteine residues) in the nuclear protein binding site of the DNA, leading to decreased GLUT-4 expression. On the other hand, *N*-acetyl cysteine (NAC) can restore the expression and translocation of GLUT-4 [4] by increasing ROS scavenging through hydrogen abstraction and glutathione replenishment [5]. Therefore, we hypothesised that the extracts of *A. saligna* could decrease the levels of ROS in the adipocytes since active extracts and their isolated constituents have reduced the activities of 2,2-di(4-*tert*-octylphenyl)-1-picrylhydrazyl and (DPPH^•^) and 2,2′-azino-bis-(3-ethylbenzothiazoline-6-sulphonic acid) (ABTS^•^*) radicals. The structural features required for antioxidants such as phenolics and flavonoids depend on the number and positions of hydroxyl (OH) groups on aromatic rings, the α,β-unsaturated carbonyl group, and other conjugated C=C double bonds in the flavonoid or phenolic structures. These structural features allow them to donate hydrogen or a single electron to a reactive free radical to form an inactive species, resulting in the termination of the damaging radical chain reactions [6].

**Figure 1:**
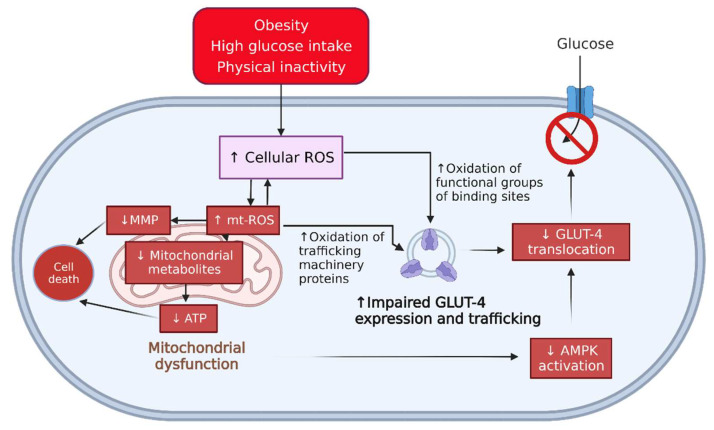
An illustrative scheme summarising the effects of excess cellular ROS and mt-ROS on the GLUT-4 in adipocytes. Obesity, a high-glucose diet, and less physical activity can increase the production of ROS. Elevated cellular ROS levels can, in turn, induce the production of mitochondrial ROS and vice versa [8], which oxidise the moiety groups of DNA binding sites for GLUT-4 transcription [3]. Moreover, the overproduction of mt-ROS leads to the oxidation of proteins involved in GLUT-4 function [7]. Reduced ATP production due to mitochondrial dysfunction can also impair the AMPK activation pathway [9]. These events can eventually lead to decreased glucose uptake. ↑ = increasing, ↓ = decreasing. ROS: reactive oxygen species; mt-ROS: mitochondrial ROS; MMP: mitochondrial membrane potential; ATP: adenosine triphosphate; AMPK: adenosine monophosphate-activated protein kinase; GLUT-4: glucose transporter 4.

A recent cell-based study [7] found that elevated mitochondrial ROS (mt-ROS) can oxidise proteins in the translocation machinery of GLUT-4, such as the GLUT-4 storage vehicle and *trans*-Golgi network. This event leads to the degradation of the structure and function of the glucose transporters. Furthermore, a cell-based study [8] showed that the increased production of mt-ROS could cause the decreased expression of essential enzymes for energy-generating pathways and the collapse of mitochondrial membrane potential (MMP), resulting in significantly reduced adenosine triphosphate (ATP) production. Consequently, the GLUT-4 translocation cannot occur due to the ATP shortage required for adenosine monophosphate-activated protein kinase (AMPK) activation, as shown in Figure 1.

As oxidative stress and impaired cellular glucose uptake commonly occur in adipocytes, studies on antidiabetic drugs typically employ 3T3-L1 adipocytes. Natural products with antioxidant properties are suggested to act as antidiabetics through multiple mechanisms, such as reducing ROS, normalising mitochondrial biogenesis and function, and activating AMPK signalling. This study aimed to determine the effects of the *A. saligna* extracts and their isolated compounds on the cellular ROS, mt-ROS levels, and MMP in 3T3-L1 adipocytes. Furthermore, the impact of the extracts and isolated compounds on glucose uptake and AMPK activation was also investigated to reflect the consequences of ROS scavenging activities on cellular glucose entry and its pathway. The antidiabetic potential of active *A. saligna* extracts and isolated compounds was evaluated in in vitro bioassays using adipose 3T3-L1 cells. This work enables us to ensure the consistency of our earlier discoveries of the in vitro antioxidant activities against free radicals [2].

## 2. Results and Discussion

### 2.1. Profile of Extracts and Isolated Compounds

The dried, ground powder (250 g) of flowers, leaves, or bark was extracted using the sequential steps of the polarity-based extraction method to provide four different extracts from each part of the plant. The outcome of the extraction is presented in Supplementary Table S1. We discovered that the methanolic extracts from the flowers (FL-MeOH), leaves (LF-MeOH), and bark (BK-MeOH) of Australian A. saligna were active in antioxidant and yeast a-glucosidase inhibitory assays. Ten compounds were isolated by column chromatography from their respective extracts, as listed in Table 1 [2]. The amount of each isolated compound is also listed in Table 1. The quantities of these compounds can assist in explaining the observed activities of the extracts in this study.

### 2.2. Viability Evaluation of the 3T3-L1 Adipocytes Treated with Isolated Compounds

We previously reported the non-toxicity of the methanolic extracts from the flowers, leaves, and bark of A. saligna against 3T3-L1 adipocytes using the 3-(4,5-dimethylthiazol-2-yl)-2,5-diphenyl-tetrazolium bromide (MTT) assay. FL-MeOH showed no toxic effects at the highest tested concentration (200 μg/mL). Similarly, LF-MeOH and BK-MeOH also showed no toxicity against 3T3-L1 adipocytes at 200 μg/mL after incubation for 72 h [2]. Further to this work, ten isolated compounds (Table 2) were screened using the same assay. At the highest tested concentration (125 µM), all compounds except naringenin **1** showed cell viability at 85% or higher. Naringenin **1** showed an increase in toxicity, with a percent cell viability of 78 ± 2% at 125 µM, while at 62.5 µM, it showed a percent cell viability of 87 ± 8%. This suggests that naringenin **1** is safe below 62.5 µM. The other compounds in Table 2 are safe for 3T3-L1 adipocytes up to 125 µM. The results of the MTT assay on 3T3-L1 adipocytes treated with four different concentrations of compounds (15.63–125 μM) for 24, 48, and 72 h are shown in Supplementary Table S2. These safe doses were evaluated in this study.

### 2.3. Measurement of Cellular ROS Level

**Figure 2:**
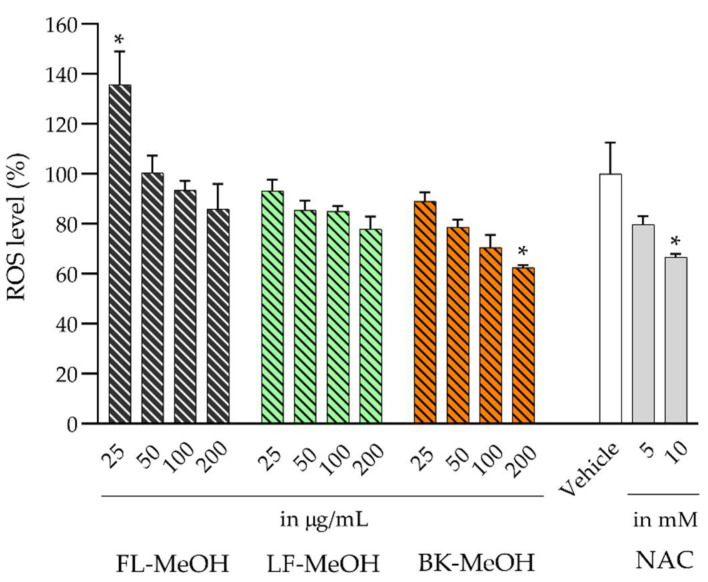
Cellular ROS levels in 3T3-L1 adipocytes treated with methanolic extracts; * *p =* 0.03, *p* was obtained from values of indicated samples vs. vehicle control (*n* = 3, one-way ANOVA, with Tukey’s post hoc tests).

The excess accumulation of lipids in the 3T3-L1 cells can lead to the overproduction of ROS due to nicotinamide adenine dinucleotide phosphate (NADPH) oxidation [10], glucose autoxidation [11] and low-density lipoprotein (LDL) peroxidation [12]. Using a 2′,7′-dichlorodihydrofluorescein diacetate (DCFH-DA) fluorescent redox probe, our study found that the cellular ROS level in 3T3-L1 adipocytes was about 50% higher than that in undifferentiated 3T3-L1 cells. All methanolic extracts demonstrated a noticeable dose-dependent reduction in ROS. As shown in Figure 2, a decreasing trend for the adipocytes’ ROS was observed for all methanolic extracts. At 25 µg/mL, LF- and BK-MeOH decreased ROS levels by 7 and 11%, respectively. In contrast, at 100 µg/mL, FL-MeOH began to reduce the ROS level. At 200 µg/mL, BK-MeOH extract significantly decreased cellular ROS level by 37% compared to the untreated cells (control). The inhibition effect of BK-MeOH on cellular ROS was slightly better than that of NAC at 10 mM. In summary, the order of effects to reduce the accumulation of ROS can be ranked as BK-MeOH > LF-MeOH > FL-MeOH. The extracts’ ability to reduce the accumulation of ROS in 3T3-L1 adipocytes reflects and follows the same trend as their in vitro antioxidant activities found in our earlier finding [2]. These findings are consistent with the study by Elansary et al., which showed a correlation between ROS-scavenging effects and the strong antioxidative activities of their methanolic leaf extract (17 μg/mL) of *A. saligna* in various cancer cell lines [13].

**Figure 3:**
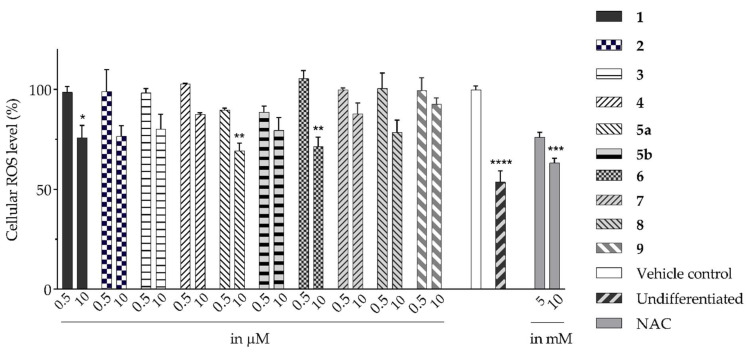
Changes in cellular ROS in 3T3-L1 adipocytes treated with isolated compounds and NAC compared to undifferentiated cells. * *p =* 0.05, ** *p =* 0.002, *** *p =* 0.0003, and **** *p =* 0.000003 were from ROS levels of the indicated samples vs. the vehicle control (*n* = 3, one-way ANOVA, with Tukey’s post hoc tests). Compound **1** = naringenin, **2** = naringenin-7-O-α-*L*-arabinopyranoside, **3** = isosalipurposide, **4** = quercitrin, **5a** = *D*-(+)-pinitol, **5b** = (−)-pinitol, **6** = (−)-epicatechin, **7** = 2,4-di-*t*-buylphenol, **8** = myricitrin, and **9** = 3-hydroxy-5-(2-aminoethyl) dihydrofuran-2(3H)-one).

Isolated compounds, such as (−)-epicatechin **6**, quercitrin **4**, and myricitrin **8**, showed potent antioxidant activities against DPPH and ABTS free radicals [2]. This suggests that the potent antioxidant activities of these compounds may also affect the accumulation of ROS and help explain the observed reduction in the accumulation of ROS by the extracts. The ability of isolated compounds to reduce cellular ROS was evaluated using the same protocol used to evaluate the extracts. Figure 3 and Supplementary Table S3 present the result of the DCFH-DA assay on the cells treated with 0.5 and 10 µM of the isolated compounds. Compared to the vehicle control, there was a reduction of about 10% in the ROS level achieved by **5a** and **5b** at 0.5 µM. In contrast, the ROS level was relatively unchanged for the other isolated compounds. A noticeable decrease was found when using 10 µM of the two compounds isolated from BK-MeOH extract, (−)-epicatechin **6** and *D*-(+)-pinitol **5a**, indicating that they were the most active compounds, with reduction percentages of 29% and 31%, respectively. *D*-(+)-pinitol **5a** demonstrated more activity than its enantiomer, (−)-pinitol **5b**, consistent with the previous finding on DPPH and ABTS scavenging [2].

Furthermore, at 10 μM, naringenin-7-O-α-*L*-arabinopyranoside **2** and 3-hydroxy-5-(2-aminoethyl) dihydrofuran-2(3H)-one **9** also reduced ROS levels by 23% and 7%, respectively. However, this finding does not reflect their weak antioxidant activities, which were observed in DPPH and ABTS assays in our previous study [2]. This inconsistency may be because compounds **2** and **9** interact with DPPH or ABTS differently compared to cellular ROS. They could possess a reductive activity toward cellular ROS via directly scavenging the ROS and were indirectly involved in cellular signalling pathways. For instance, flavanone, the core skeleton of compound **2** (Table 1), scavenged hydroxyl and peroxide radicals attributed to the hydroxyl group at C4’ of ring B [14]. Moreover, naringenin has been reported to reduce oxidative stress and improve mitochondrial function via the modulation of endogenous glutathione [15] and the activation of the nuclear factor erythroid 2-related factor 2 (Nrf2) pathway [16].

Compound **2** showed similar effects to naringenin **1** (1% and 24%) with respect to reducing ROS at both tested concentrations (1% and 23% at 0.5 and 10 µM). The glycosylated 7-OH of ring A can decrease anti-ROS activity [17]. The chalcone derivative, isosalipurposide **3**, exerted a lower level of activity (20% at 10 µM) than naringenin **1** and its glycoside derivative **2**. Saturated C2 and C3 and the absence of ring C in chalcones, such as in isosalipurposide **3**, have been suggested as the cause of its decreased capacity to inhibit ROS compared to flavanone [17].

Flavanol derivatives, including (−)-epicatechin **6,** have been reported to possess better ROS-scavenging activities than many monomeric flavanones and flavonols [2]. Our DPPH and ABTS assays showed that (−)-epicatechin **6** exerted better inhibition effects than flavanone derivatives, including naringenin **1** and naringenin-7-O-α-*L*-arabinopyranoside **2**, and flavonol derivatives such as quercitrin **4** and myricitrin **8** [2]. Furthermore, the mechanism of action reported by Verri Jr. et al. [18] on the cell signalling system plays a key role in the protective effect of bioactive compounds against overproduced ROS. We found reductions of 29%, 21%, and 12% in ROS levels at 10 µM for (−)-epicatechin **6,** myricitrin **8**, and quercitrin **4**, respectively.

### 2.4. Measurement of Mt-ROS and MMP on Adipocytes

Given that accumulated mt-ROS can trigger carbonylation in mitochondrial proteins and damage the antioxidant enzymes, such as the superoxide dismutase of manganese superoxide dismutase (MnSOD) and superoxide dismutase (SOD2), the reduction of mt-ROS can restore mitochondrial function. An increase in the MMP can manifest the restored mitochondria. An increased MMP has been reported to improve endogenous mitochondrial antioxidants to convert superoxide into harmless H_2_O [19].

Changes in the MMP can be monitored using a membrane-permeant JC-1 dye assay. In the assay, the cationic lipophilic JC-1 dye accumulates in polarised mitochondria to form a J-aggregate that fluoresces red. JC-1 leaves the inner mitochondrial membrane upon depolarisation for the cytoplasm, where it disaggregates into monomers (JC-1 monomer) that show green fluorescence. The degree of depolarisation is determined by the ratio of red/green fluorescence or J-aggregates/JC-1 monomers. A healthy mitochondrion possesses more negative charges in the mitochondrial matrix due to proton transfer from the matrix to intermembrane space. Therefore, healthy mitochondria admit red fluorescence compared to mitochondria with a lower membrane potential, which fluoresce green. In other words, it has a higher red/green ratio [20].

Our study showed that the mt-ROS level was 30% lower and the ratio of J-aggregates/JC-1 monomers was 91% higher in undifferentiated 3T3-L1 cells than in those measured for the adipocytes. In addition, treatment with a mitochondria-targeting drug, metformin, demonstrated a reduction in mt-ROS of 34% and a 2-fold higher ratio of J-aggregates/JC-1 monomers than the vehicle control. Thus, these parameters can reflect the normalisation of the mitochondrial function. Metformin was used as a positive control instead of NAC in studying mitochondrial function as it explicitly targets the primary source of mt-ROS for signalling pathways and communication between the mitochondria and the rest of the cell, the reverse electron transfer (RET) sites at complex I of the mitochondrial electron transfer chain. Instead of scavenging entire ROS that can alter the proportional ROS level, leading to deteriorating pathological effects [21], it selectively neutralises the excess superoxides produced by RET without interfering with the generation of ROS via the forward direction [22]. This modulates the effects of MMP restoration and AMPK-α phosphorylation [23].

As presented in Table 3, exposure to FL-, LF-, and BK-MeOH extracts at the higher concentration (50 µg/mL) positively impacted mitochondrial health in adipocytes, indicated by the decrease in the mt-ROS levels and the increase in the ratios of J-aggregates/JC-1 monomers compared to untreated adipocytes. All methanolic extracts demonstrated a reducing effect on mt-ROS levels at 50 μg/mL. The methanolic flower (FL-MeOH) extract reduced the levels of mt-ROS by 31%, while the ROS levels were estimated to be reduced by 53% and 58% by LF- and BK-MeOH, respectively. Treatment with FL-MeOH increased the MMP by 2-fold, while treatment with BK- and LF-MeOH increased the MMP by 1.47- and 0.79-fold, respectively.

The decreasing trend of the mt-ROS levels was also displayed in the isolated compounds (Table 4). At 10 μM, all compounds except compound **9** showed reductions in mt-ROS levels by 28 to 54%. The poor inhibitory effect of lactone **9** is consistent with its poor antioxidant activity [2]. (−)-Epicatechin **6** demonstrated significant effects at 5 and 10 μM, indicating that this flavanol possesses strong antioxidant properties. This finding aligns with the marked inhibitory activities against DPPH^•^ and ABTS^•^* radicals, suggesting an antioxidant capacity.

At 10 μM, naringenin **1**, D-(+)-pinitol **5a**, (−)-pinitol **5b**, and (−)-epicatechin **6**, demonstrated significant protective effects on the mitochondria of the adipocytes that were similar or better than metformin. D-(+)-pinitol **5a** showed potent effects of mitochondrial protection. It is 1.5-fold stronger than metformin at the same concentration of 10 μM. D-(+)-pinitol **5a** has been shown to protect mitochondria by increasing intracellular glutathione and an endogenous antioxidant of glutathione reductase in P12 cells [24]. Notably, D-(+)-pinitol **5a** has been used as a natural health supplement to provide therapeutic benefits in treating T2D as an insulin regulator [25]. It also has anti-inflammatory [25] and hepatoprotective [26] activities.

Apart from their role in the protein signalling pathway, flavonoids are also believed to modulate levels of endogenous antioxidant enzymes such as superoxide dismutase, catalase, glutathione peroxidase, and glutathione-S-transferase [27]. Notably, naringenin **1** and (–)-epicatechin **6** demonstrated outstanding inhibition of mt-ROS and protection of mitochondria. In studies using human vascular endothelial cells (HUVECs), (–)-epicatechin **6** has been shown to alter the production of mt-ROS under stress [28] through activating AMPK and the silent information regulator 1 (SIRT1) signalling pathway [29].

Here, we showed that the mitochondrial health of adipocytes was enhanced by the methanolic extracts of A. saligna and its isolated phytochemicals. This was confirmed by the comparison of data regarding the mt-ROS levels and the increase in the ratios of J-aggregates/JC-1 monomers with those observed from the treatment with metformin and undifferentiated 3T3-L1 cells.

### 2.5. Cellular Glucose Uptake Assay

**Figure 4:**
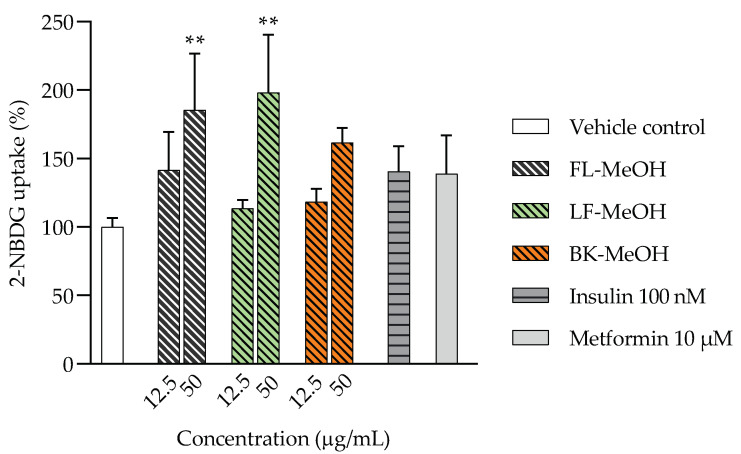
2-NBDG uptake by 3T3-L1 adipocytes treated with extracts of *A. saligna.* ** *p =* 0.007 for FL-MeOH and ** *p =* 0.006 for LF-MeOH compared to the vehicle control (*n* = 3, one-way ANOVA, with Dunnett’s post hoc tests).

Treatment with methanolic extracts (FL-MeOH, LF-MeOH, and BK-MeOH) and isolated compounds (Figure 1) reduced ROS and protected mitochondria in 3T3-L1 adipocytes, so we postulated that they might also stimulate cellular glucose uptake. The 2-(*N*-(7-nitrobenz-2-oxa-1,3-diazol-4-yl)-amino)-2-deoxyglucose (2-NBDG) assay was performed to evaluate glucose uptake. All extracts showed a dose-dependent increase in glucose uptake at 12.5 and 50 μg/mL. Marked increases in glucose uptake of 98% and 85% were observed when cells were treated with 50 μg/mL LF-MeOH and FL-MeOH, respectively. At 50 μg/mL FL-, LF-, and BK-MeOH performed better than 10 μM of metformin. The glucose uptake potency of the extracts can be ranked as LF-MeOH > FL-MeOH > BK-MeOH, as shown in Figure 4 and Supplementary Table S4.

**Figure 5:**
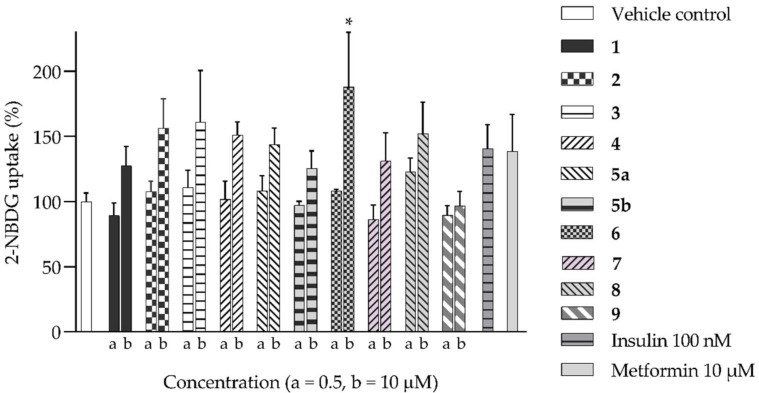
2-NBDG uptake by 3T3-L1 adipocytes treated with isolated compounds. * *p =* 0.01, *p* value was from the indicated sample against the vehicle control (*n* = 3, one-way ANOVA, with Dunnett’s post hoc tests). a = 0.5 µM, b = 10 µM. Compound **1** = naringenin, **2** = naringenin-7-O-α-*L*-arabinopyranoside, **3** = isosalipurposide, **4** = quercitrin, **5a** = *D*-(+)-pinitol, **5b** = (−)-pinitol, **6** = (−)-epicatechin, **7** = 2,4-di-*tert*-buylphenol, **8** = myricitrin, and **9** = 3-hydroxy-5-(2-aminoethyl) dihydrofuran-2(3H)-one).

We further evaluated the isolated compounds (Table 1) for their ability to improve glucose uptake in 3T3-L1 adipocytes to determine which compounds were responsible for stimulating the glucose uptake observed in the active extracts. The outcome is reported in Figure 5 and Supplementary Table S5. A slightly positive enhancement in the uptake stimulation was observed in the treatment at 0.5 μM by compound **2** (8% increase), isosalipurposide **3** (11%), *D*-(+)-pinitol **5a** (9%), (−)-epicatechin **6** (8%), and myricitrin **8** (23%) compared to the vehicle control. At 10 μM, an increase in 2-NBDG uptake was observed across all compounds (except compound **9**). Interestingly, (−)-epicatechin **6** (88% increase) performed the best and better than metformin. Other compounds, such as compounds **2** (56%) and **3** (61%), quercitrin **4** (51%), **5a** (44%), **7** (31%) and myricitrin **8** (52%), showed marked improvements in glucose uptake. Therefore, the effects of isolated compounds on glucose uptake support the activities of their respective extracts.

Impaired mitochondrial function in white adipose tissue can lower cellular glucose uptake due to insulin resistance [30]. Here, we demonstrated that the most active extract, LF-MeOH at 50 µg/mL, resulted in a 98% increase in glucose uptake, a 53% reduction in mt-ROS, and 1.8-times increase in the J-aggregates/JC-1 monomers ratio. At 10 µM, (−)-epicatechin **6** increased glucose uptake by 88%, reduced mt-ROS levels by 54%, and provided a 2.3-times increase in the J-aggregates/JC-1 monomers ratio. This finding suggests that *A. saligna* methanolic extracts and compounds can protect adipocyte mitochondria and consequently enhance cellular glucose uptake.

### 2.6. Immunoblot Analysis of AMPK Pathway Activation

**Figure 6:**
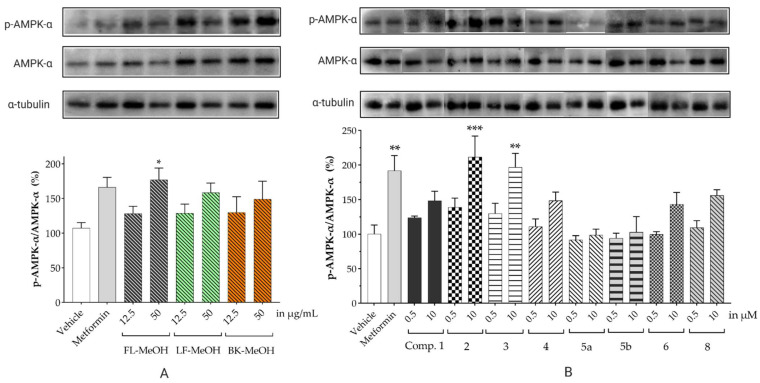
The ratio of expressed p-AMPK-α to AMPK-α in 3T3-L1 adipocytes treated with MeOH extracts (**A**) and isolated compounds (**B**). Data in mean ± SEM; * *p =* 0.02; ** *p =* 0.003, *** *p =* 0.0002, *p* values were from values of indicated samples vs. the vehicle control (*n* = 3, one-way ANOVA, with Tukey’s post hoc tests). Comp., **1** = naringenin, **2** = naringenin-7-O-α-*L*-arabinopyranoside, **3** = isosalipurposide, **4** = quercitrin, **5a** = *D*-(+)-pinitol, **5b** = (−)-pinitol, **6** = (−)-epicatechin, and **8** = myricitrin).

A signalling pathway linking mitochondrial function to enhanced glucose uptake in the differentiated 3T3-L1 cells treated with all methanolic extracts was evaluated using Western blot analysis. The study focused on the activation of AMPK signalling as this pathway has been reported to regulate mitochondrial function and GLUT4 translocation. Figure 6A, Supplementary Figure S1, and Table S6 show the elevation of the phosphorylation of the AMPK-α subunit (p-AMPK-α) in a dose-dependent manner by the extracts. These results suggest an increased activation of AMPK-α. FL-MeOH at 50 μg/mL showed the highest expression of p-AMPK-α among the three extracts. There was a correlation between the improvement in glucose uptake (Figure 4) and increases in the phosphorylation of AMPK-a when treated with 50 μg/mL of the extracts. For instance, FL-MeOH increased glucose uptake and p-AMPK-a phosphorylation by 85% and 77%, respectively.

The possible link between glucose uptake and the AMPK signalling pathway of the isolated compounds was also evaluated. Due to their marginal glucose uptake, 2,4-di-*tert*-buylphenol **7** and 3-hydroxy-5-(2-aminoethyl) dihydrofuran-2(3H)-one) **9** were not assessed for activation of the AMPK signalling pathway. For other isolated compounds, at 10 μM of the compounds, we observed enhanced AMPK-α phosphorylation across all compounds (except **5a**), as shown in Figure 6B and Supplementary Figures S1–S3 and Table S7. A marked increase in p-AMPK-α was observed for naringenin **1** (48%), naringenin-7O-α-L-arabinopyranoside **2** (112%), isosalipurposide **3** (97%), quercitrin **4** (49%), (−)-epicatechin **6** (43%), and myricitrin **8** (56%). The highest level of p-AMPK-α was observed in the test of naringenin-7O-α-L-arabinopyranoside **2** at 10 μM, which was shown to improve glucose uptake in 3T3-L1 adipocytes (Figure 5). This finding suggests that they stimulate glucose uptake via AMPK-a phosphorylation. D-(+)-pinitol **5a** improved glucose uptake; however, its ability to activate AMPK-a was not observed at 10 μM. D-pinitol **5a** is well known to increase glucose-induced insulin secretion by reducing the expression of AMPK-α [31].

As previously mentioned, excessive levels of mt-ROS can decrease the MMP in adipocytes, disrupting the cellular uptake of glucose and the AMPK-α. The decrease in p-AMPK-α has been suggested as the primary cause of mitochondrial dysfunction due to the impaired activation of oxidative phosphorylation in the mitochondria [30]. The reduced activation of AMPK signalling is also thought to be the consequence of mitochondrial dysfunction initiated by the obesity-induced overproduction of ROS.

*A. saligna* methanolic extracts and the isolated compounds are promising inhibitors of the production of cellular ROS and mt-ROS. Furthermore, the extracts and the compounds markedly increased glucose uptake and p-AMPK-α levels in 3T3-L1 adipocytes. The activation of the AMPK pathway has also been linked to the reduced production of cellular ROS and mt-ROS in 3T3-L1 adipocytes [3] (Figure 1). In addition, this pathway can protect the downstream target GLUT-4 from structural degradation and dysfunction due to excessive levels of ROS [3,4,32]. These findings suggest that active glucose transporters can be activated by the AMPK pathway to facilitate glucose entry into fat cells.

## 3. Materials and Methods

### 3.1. Materials

The solvent for extraction included *n-*hexane, dichloromethane (DCM), and methanol (MeOH) (Point of Care Diagnostics, North Rocks, NSW, Australia). Ultrapure water was purified using an Aurium pro-VF ultrapure water system (Gottingen, Germany). The 3T3-L1 fibroblast cell lines were purchased from American Type Tissue Culture/ATCC (Manassas, VA, USA). Unless otherwise expressed, all chemicals were purchased from Sigma-Aldrich (St. Louis, MO, USA). The following reagents were used for cell culture: Dulbecco’s Modified Eagle Medium (DMEM) high glucose, bovine calf serum (BCS), penicillin, streptomycin glutamine (PSG), foetal bovine serum (FBS), rosiglitazone, dexamethasone, 3-isobutyl-1-methylxanthine (IBMX), insulin, phosphate-buffered saline (PBS), trypsin-EDTA solution 0.25%, bovine serum albumin (BSA), and dimethylsulfoxide (DMSO). The chemicals used for cell-based assays were dichlorodihydrofluorescein diacetate (DCFH-DA), (3-(4,5 dimethylthiazol-2-yl)-2, 5 diphenyltetrazolium bromide) (MTT), metformin, Hank’s balanced salt solution (HBSS), *N*-acetylcysteine (NAC), metformin, 5,5,6,6′-tetrachloro-1,1′, 3,3′ tetraethylbenzimi-dazoylcarbocyanine iodide (JC-1), insulin, and bovine serum albumin (BSA). A Krebs–Ringer phosphate HEPES (KRPH) buffer was prepared from NaCl 118 mM, KCl 5 mM, KH_2_PO_4_ 1.2 mM, CaCl_2_ 1.3 mM, MgSO_4_ 1.2 mM, and HEPES 30 mM in a specific volume of milli-Q water. The 2-deoxy-2-[(7-nitro-2,1,3-benzoxadiazol-4-yl) amino]-*D*-glucose (2-NBDG) and hydroethidine triphenylphosphonium cation (MitoSOX) were purchased from Thermo-Fisher Scientific (Eugene, OR, USA).

The following materials were supplied by Merck (Darmstadt, Germany): polyvinylidene fluoride membranes, skim milk powder, Immobilon ECL Ultra Western HRP substrate, RIPA lysis buffer, glycine, Bradford reagent, hydrochloric acid and sodium dodecyl sulfate (SDS). The other materials were Tween-20 (Vivantis Inc., Oceanside, CA, USA), Tris (Vivantis Inc., Oceanside, CA, USA), ammonium peroxide sulphate, sodium hydroxide, protein and ladder standard solution, acrylamide/bis-acrylamide solution 30% (HiMedia Laboratories, India), tetramethylethylenediamine or TEMED (PanReac AppliChem & ITW Reagents, Darmstadt, Germany), and Laemmli sample buffer (BioRad Laboratories, Hercules, CA, USA). The following materials were purchased from Cell Signalling Technology (Danvers, MA, USA): protease inhibitor cocktail, primary antibodies (p-AMPK-α, AMPK-α, and α-tubulin), and goat anti-rabbit IgG horseradish peroxidase-conjugated (HRP-conjugated) secondary antibody.

### 3.2. Sample Collection, Extraction, Compound Isolation, and Molecular Elucidation

The samples, including the leaves, flowers, and stem bark, were collected from 12 Tasman Street, Kurnell, Sutherland Shire, NSW (34°00′48.2″ S 151°12′27.7″ E) on 7 October 2019. The taxonomy of the plant was determined by Andrew Orme (voucher number BIS 21186), a technical identification officer from the National Herbarium of NSW, as *Acacia saligna* (Labill.) H.L.Wendl. In addition, the plant was further classified into the informal subsp. saligna by Bruce Maslin from the Western Australia Herbarium. All the dried samples were successively extracted with *n-*hexane, DCM, MeOH, and water. Furthermore, the details of the compound separation, molecular elucidation, and spectral information of the active compounds isolated from methanolic extracts of *A. saligna* were presented in our previous report [2].

### 3.3. Cell Culture and Differentiation

The cells were cultivated in a tissue culture flask (Corning, New York, NY, USA) at a density of 0.3 × 10^5^ cells/mL in a complete growth medium of DMEM (90%, *v*/*v*) supplemented with BCS (9%) and PSG (1%). The cells were incubated in a humid environment of 37 °C with 5% CO_2_ and harvested at 70–80% confluent. The pre-confluent cells were then seeded on a 96-multiwell plate (3 × 10^3^ cells/mL each well) and incubated with the same complete medium for 4 days to reach confluence. On the day of differentiation induction (set as day 0), the confluent cells were exposed to a medium of differentiation induction or MDI (90% DMEM, 9% FBS, 1% PSG, rosiglitazone 2 µM, dexamethasone 2.2 mM, IBMX 500 mM, and insulin 4 mg/mL), followed by incubation for 48 h. The MDI was replaced by a differentiation-maintaining medium (90% DMEM, 9% FBS, 1% PSG, and insulin 4 mg/mL) for a further 48 h of incubation. The medium was replaced every 2 days. The cells were then exposed to the basal medium (90% DMEM, 9% FBS, and 1% PSG) on day 6 and incubated for 48 h to obtain mature, differentiated cells.

### 3.4. Viability of 3T3-L1 Adipocytes

The 3T3-L1 adipocytes seeded in three different 96-well plates were incubated with basal medium containing extracts of the flowers, leaves, and bark of *A. saligna* in various concentrations (25–200 µg/mL) or isolated compounds (15.63–125 µM) for 24, 48, and 72-h. The cells were washed with PBS and then exposed to a fresh basal medium with 10% MTT (5 mg/mL dissolved in PBS). After incubation for another 4 h, the supernatant was replaced by 100 μL of DMSO and mixed correctly. The absorbance was measured using a microplate reader (Tecan Infinite M1000 PRO, Männedorf, Switzerland) at 570 nm. The viable cell was calculated via the following formula:

$$\mathrm{Cell}\mathrm{viability}(\%)=\left(\frac{\mathrm{Absorbance}\mathrm{of}\mathrm{treated}\mathrm{adipocytes}}{\mathrm{Absorbance}\mathrm{of}\mathrm{vehicle}\mathrm{control}}\right)\times 100$$

### 3.5. Determination of Cellular ROS Production

The ROS detection experiment via DCFH-DA assay in 96-well polystyrene black microplates (Corning, New York City, NY, USA) was adapted from a published protocol [33]. On day 8 of the differentiation, the adipocytes were incubated for 48 h with 100 µL of fresh basal medium for the vehicle control (DMSO 0.1%), *N*-acetylcysteine (NAC) solution (5 and 10 mM) as the positive control, and the extract-containing medium (25–200 μg/mL) or isolated-compounds-containing medium (15.63–125 µM). After discarding the supernatants, the cells were gently washed with PBS, 10 µM of DCFH-DA solution (100 µL) was added, and the cells were covered with aluminium foil and then incubated for 45 min. Afterwards, the supernatants were removed, the cells were washed with Hank’s balanced salt solution (HBBS), and then HBBS was added. The intensity of fluorescence corresponding to the concentration of cellular ROS was read at excitation and emission wavelengths of 485 and 530 nm, respectively, with a plate fluorescence reader. The percentage of the ROS level is calculated by:

$$\mathrm{ROS}\mathrm{level}(\%)=\left(\frac{\mathrm{Fluorescence}\mathrm{of}\mathrm{treated}\mathrm{adipocytes}}{\mathrm{Fluorescence}\mathrm{of}\mathrm{vehicle}\mathrm{control}}\right)\times 100$$

### 3.6. Cellular Glucose Uptake Assay

A glucose uptake simulation was performed using a 2-NBDG fluorescent assay in 96-well black plates, adapted from a published protocol [34]. The mature differentiated cells were serum-starved overnight under humidified conditions with low-glucose DMEM and BSA 0.1%. After medium removal, the further incubation of the adipocytes for 1 h was carried out with the KRPH buffer solution. After another incubation for 30 min at 37 °C followed with the following treatments: extracts (12.5 and 50 µg/mL) or isolated compounds (0.5 and 10 µM); vehicle medium (vehicle control); and insulin (100 nM) and metformin (10 µM) as positive controls 1 and 2, respectively; all were dissolved in the KRPH buffer (100 μL/well), and another identical volume of KRPH buffer containing 2-NBDG (80 μM) was added into each well, followed by further incubation for 30 min. After removing the medium and washing with ice-cooled PBS, the fluorescence was measured using a PerkinElmer microplate reader (Waltham, MA, USA) at excitation (λ_ex_) and emission wavelengths (λ_em_) of 485 and 535 nm, respectively. The glucose uptake values were expressed by:

$$\mathrm{Glucose}\mathrm{uptake}(\%)=\left(\frac{\mathrm{Fluorescence}\mathrm{of}\mathrm{treated}\mathrm{adipocytes}}{\mathrm{Fluorescence}\mathrm{of}\mathrm{vehicle}\mathrm{control}}\right)\times 100$$

### 3.7. Mt-ROS Level Measurement

The modified protocol from Kauffman et al. [35] was adapted for the experiment of the mt-ROS probe using the mitoSOX in the 96-well black plates. The mature 3T3-L1 adipocytes were incubated for 24 h with a basal medium containing DMSO 0.1% and methanolic extracts (12.5 and 50 µg/mL) or isolated compounds (0.5, 5, and 10 µM). The basal medium (vehicle medium) was used as the blank control, while metformin (10 µM) was a positive control. Following the incubation and the discarding of the medium, the treated mature adipose cells were then exposed to 5 µM of MitoSOX Red in 100 µL of PBS to tag the mt-ROS, covered with aluminium foil, and incubated for 30 min. Afterwards, the cells were washed with PBS twice, and 100 µL of PBS was added. The fluorescence was read with the PerkinElmer microplate reader (Waltham, MA, USA) at λ_ex_ 510 and λ_em_ 580 nm. The obtained data were expressed in percentage of the observed parameter:

$$\mathrm{Mt}-\mathrm{ROS}\mathrm{level}(\%)=\left(\frac{\mathrm{Fluorescence}\mathrm{of}\mathrm{treated}\mathrm{adipocytes}}{\mathrm{Fluorescence}\mathrm{of}\mathrm{vehicle}\mathrm{control}}\right)\times 100$$

### 3.8. Mitochondrial Membrane Potential (MMP) Measurement

The MMP measurement via JC-1 was carried out in the 96-multiwell black plates, following Harshkova et al. [36] with a slight modification. Similar to the procedure described for labelling the mt-ROS, after 24 h of treatment with the methanolic extracts or isolated compounds, blank control, and positive control of metformin, the mitochondria of the adipocytes were stained with 3 µM of JC-1 for 30 min in a humidified incubator. After washing with PBS twice and adding PBS (100 µL), the fluorescent optical density (OD) was read at λ_ex_ 488 nm and λ_em_ 530 nm (green JC-1 monomers) and λ_ex_ 443 nm and λ_em_ 590 nm (red J aggregates). The obtained data were converted into a ratio of J-aggregates to JC-1 monomers (R). The MMP was expressed as a percentage of the ratio of J-aggregates to JC-1 monomers for samples (Rs) normalised by the ratio for the vehicle control (Rc).

$$J-\mathrm{aggregates}\mathrm{to}\mathrm{JC}-1\mathrm{monomers}\mathrm{ratio}(\%\mathrm{of}\mathrm{control})=\frac{\mathrm{Rs}}{\mathrm{Rc}}\times 100$$

### 3.9. The Immunoblot Analysis

The immunoblot analysis protocol was adapted from Lee et al. [37] with a minor change. After treatment with the indicated agents, the cells were washed in ice-chilled PBS, harvested via 100 μL of lysis buffer, and spun for 10 min at 12,000 rpm at 4 °C. The Bradford-based protein content was determined out using BSA as the standard, with a range of concentration of 0.0375 to 6 mg/mL in a 96-well plate. The lysates were then heated at 95 °C for 10 min and centrifugation at 5000 rpm for 2 min. Electrophoresis of supernatant containing 20 μg of proteins was performed using SDS-polyacrylamide (10%) gels, followed by the electrophoretic transfer of the separated proteins to PDVF membranes. Afterwards, the membranes were blocked with 5% non-fat milk solution for 1 h and then incubated with the appropriate primary antibody solutions at a dilution of 1:1000 overnight at 4 °C. After washing with Tris-buffered saline and Tween-20 (0.1%), the membranes were incubated with goat anti-rabbit IgG horseradish peroxidase-conjugated (HRP-conjugated) secondary antibodies (1:10,000) for one hour at room temperature. Bands were then visualised with ECL Western blotting reagents, and the chemiluminescent images were captured using a Chemidoc system (Amersham Image-quant 800, Marlborough, MA, USA). A stripping step was performed before re-probing with other antibodies (total AMPK and α-tubulin). The captured bands were analysed using Image J (National Institute of Health, Bethesda, MD, USA) to quantify the protein expression.

### 3.10. Statistical Analysis

Data are expressed as mean ± standard error mean (SEM) from the three separate experiments (*n* = 3). A one-way ANOVA of Dunnett’s or Tukey’s multiple comparison tests was selected to calculate the difference between the two means of each sample and the vehicle control optical density; *p* < 0.05 was considered significant (GraphPad Prism 9, San Diego CA, USA).

## 4. Conclusions

In the present work, the abilities of the *A. saligna* extracts and their isolated compounds to reduce cellular ROS and mt-ROS were consistent with their in vitro antioxidant activities against DPPH^•^ and ABTS^•^* radicals shown in our earlier report. This study demonstrated that all three methanolic extracts from the flowers (FL-MeOH), leaves (LF-MeOH), and bark (BK-MeOH) of *A. saligna* and their isolated compounds possessed antihyperglycemic properties, as shown by the improved glucose uptake, decreased oxidative stress, and maintenance of mitochondrial function in 3T3-L1 adipocytes. The LF-MeOH and BK-MeOH extracts and their isolated compound (−)-epicatechin **6** performed better than others in reducing cellular ROS and mt-ROS and maintaining mitochondrial function. Our study also showed a consistent correlation between the improvement in glucose uptake and increases in the phosphorylation of AMPK-a in 3T3-L1 adipocytes when treated with extracts and their corresponding isolated compounds. The markedly increased glucose uptake by (−)-epicatechin **6**, quercitrin **4**, and myricitrin **8** reflected the best glucose uptake of their corresponding LF-MeOH. FL-MeOH showed the highest expression of p-AMPK-α among the three extracts. These results were supported by the marked increase in p-AMPK-α when treated with isolated compounds such as naringenin **1**, naringenin-7-O-α-L-arabinopyranoside **2**, isosalipurposide **3**, quercitrin **4**, (−)-epicatechin **6**, and myricitrin **8**. Among these compounds, (−)-epicatechin **6** performed well for all tested activities and significantly increased the levels of phosphorylated AMPK-α, suggesting that its effects could be partly mediated via the activation of the AMPK signalling pathway. Our findings suggest that *A. saligna* extracts and their isolated compounds may have an antihyperglycemic effect that could be useful for further developing new treatments for diabetes.

## Figures and Tables

**Table 1:** Isolated compounds from the methanolic flower (FL-MeOH), leaf (LF-MeOH), and bark (BK-MeOH) extracts of A. saligna [2].

Compounds	Extract/Amount (%*w*/*w* Extract)	Compound	Extract/Amount (%*w*/*w* Extract)
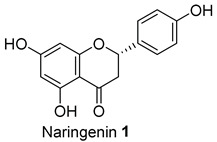	FL-MeOH/1.75	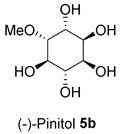	LF-MeOH/8
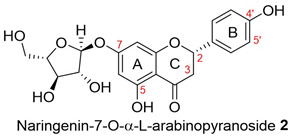	FL-MeOH/2.58	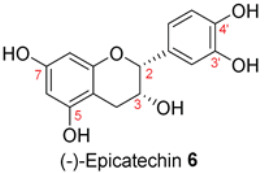	LF-MeOH and BK-MeOH/0.9 ^b^, 2.53 ^c^
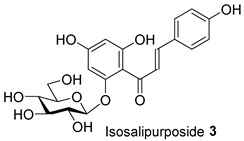	FL/MeOH/1.52	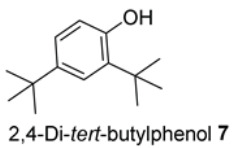	LF-MeOH/1
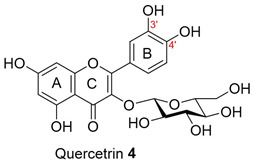	FL-MeOH and LF-MeOH/4.13 ^a^, 2.68 ^b^	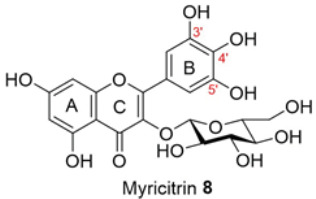	LF-MeOH/5
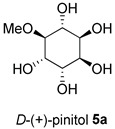	FL-MeOH and BK-MeOH/2.5 ^a^, 17.83 ^c^	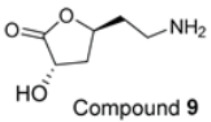	LF-MeOH/5

^a^ from FL-MeOH, ^b^ from LF-MeOH; ^c^ from BK-MeOH.

**Table 2:** Cell viability percentage of 3T3-L1 adipocytes exposed to isolated compounds for 72-h.

Compound	% Viability of 3T3-L1 Adipocytes at Concentrations of (µM)			
	15.63	31.25	62.5	125
Naringenin **1**	93 ± 6	85 ± 6	87 ± 8	78 ± 2 **
Compound **2**	78 ± 4 *	79 ± 6 *	90 ± 6	90 ± 5
Isosalipurposide **3**	100 ± 4	101 ± 4	101 ± 4	101 ± 3
Quercitrin **4**	99 ± 1	99 ± 2	99 ± 2	100 ± 2
*D*-(+)-pinitol **5a**	98 ± 5	99 ± 4	98 ± 3	98 ± 3
(–)-Pinitol **5b**	94 ± 3	94 ± 4	94 ± 4	96 ± 4
(–)-Epicatechin **6**	89 ± 7	95 ± 2	92 ± 1	87 ± 7 *
2,4-Di-*tert*-butylphenol **7**	99 ± 1	82 ± 5 *	89 ± 11	90 ± 8
Myricitrin **8**	105 ± 5	105 ± 5	103 ± 3	104 ± 4
Compound **9**	83 ± 3 *	96 ± 3	90 ± 6	85 ± 5 *
Vehicle	100 ± 1			

* *p =* 0.01, ** *p* = 0.003, *p* values were from samples against the vehicle control (*n* = 3, one-way ANOVA, with Dunnett post hoc tests).

**Table 3:** Changes in mt-ROS levels and J-aggregates/JC-1 monomers ratios in 3T3-L1 adipocytes treated with methanolic extracts.

Sample	Mt-ROS Level (%)		J-Aggregates (Red)/JC-1 Monomers (Green) Percentage (%)	
	12.5 μg/mL	50 μg/mL	12.5 μg/mL	50 μg/mL
Vehicle control	100 ± 9		100 ± 10	
FL-MeOH	103 ± 28	68 ± 27	138 ± 24	304 ± 8 ***
LF-MeOH	98 ± 9	47 ± 20	97 ± 2	179 ± 19
BK-MeOH	103 ± 24	42 ± 4	124 ± 33	247 ± 29 *
Metformin 10 µM	67 ± 2		207 ± 42	
Undifferentiated cells	71 ± 8		191 ± 11	

Data expressed as mean ± SEM, * *p* = 0.03, *** *p* = 0.0002, *p* values were from the value of indicated samples vs. vehicle control (*n* = 3, one-way ANOVA, with Tukey’s post hoc tests).

**Table 4:** Estimated mt-ROS levels and J aggregates/JC-1 monomers ratios in 3T3-L1 adipocytes treated with isolated compounds.

Sample	Mt-ROS Level (%)			J Aggregates/JC-1 Monomers Percentage (%)		
	0.1 μM	5 μM	10 μM	0.1 μM	5 μM	10 μM
Vehicle control	100 ± 9			100 ± 10		
Naringenin **1**	65 ± 6	65 ± 8	49 ± 11 **	95 ± 3	173 ± 13	267 ± 31 ****
Compound **2**	72 ± 3	69 ± 3	57 ± 6 *	107 ± 10	124 ± 17	206 ± 11 *
Isosalipurposide **3**	78 ± 2	64 ± 5	57 ± 7 *	89 ± 7	127 ± 8	164 ± 6
Quercitrin **4**	91± 2	72 ± 9	57 ± 4 *	74 ± 12	105 ± 21	128 ± 7
D-(+)-pinitol **5a**	71 ± 7	72 ± 3	56 ± 8 *	121 ± 20	142 ± 16	301 ± 42 ****
(−)-Pinitol **5b**	103 ± 4	64 ± 5	55 ± 3 *	196 ± 28	224 ± 29*	238 ± 21 **
(−)-Epicatechin **6**	67 ± 7	56 ± 10 *	46 ± 4 **	119± 13	120 ± 18	225 ± 32 **
2,4-Di-tert-buylphenol **7**	73 ± 1	68 ± 7	46 ± 8 **	92 ± 5	148 ± 24	165 ± 9
Myricitrin **8**	84 ± 15	70 ± 17	68 ± 5	127 ± 14	132± 22	194 ± 14
Compound **9**	95 ± 18	89± 7	78 ± 8	148 ± 25	135 ± 15	187 ± 23
Metformin 10 µM	66 ± 2			207 ± 42		
Undifferentiated cells	71 ± 8			191 ± 11		

Data expressed as mean ± SEM, * *p* = 0.03, ** *p* = 0.001, **** *p* = 0.000001, *p* values were obtained from values of indicated samples vs. vehicle control (*n* = 3, one-way ANOVA, with Tukey’s post hoc tests).

## Data Availability

Data are contained within the article.

## Supplementary Materials

The following supporting information can be downloaded at: https://www.mdpi.com/article/10.3390/molecules28104054/s1, Table S1. The extraction results from dried (250 g) flowers, leaves, or bark of *A. saligna*; Table S2. Viable 3T3-L1 preadipocytes and adipocytes treated with isolates for 24, 48, and 72 h; Table S3. The estimated ROS level of adipocytes exposed to isolated compounds for 48 h; Table S4. Observed data of glucose uptake simulation with the fluoroprobe 2-NBDG assay for all extracts on the 3T3-L1 adipocytes; Table S5. Observed data of glucose uptake simulation with 2-NBDG fluorescence assay for isolated compounds on the 3T3-L1 adipocytes; Table S6. Quantitative data of the ratio of p-AMPK-α to AMPK-α (%) expressed by adipocytes exposed to the tested MeOH extracts; Table S7. Quantitative data of the ratio of p-AMPK-α to AMPK-α (%) expressed by adipocytes exposed to the tested isolates; Figure S1a–c. Original Western blot images of membrane 1 for the immunoblot analysis (lane **1**: vehicle control, **2**: metformin 10 μM, **3**: FL-MeOH 12.5 μg/mL, **4**: FL-MeOH 50 μg/mL, **5**: LF-MeOH 12.5 μg/mL, **6**: LF-MeOH 50 μg/mL, **7**: BK-MeOH 12.5 μg/mL, **8**: BK-MeOH 50 μg/mL, **9**: compound **2** 0.5 μM, **10**: compound **2** 10 μM, **11**: isosalipurposide **3** 0.5 μM, and **12**: isosalipurposide **3** 10 μM); Figure S2a–c. Original Western blot images of membrane 2 for the immunoblot analysis (lane **1**: vehicle control, **2**: metformin 10 μM, **3**: quercitrin **4** 0.5 μM, **4**: quercitrin **4** 10 μM, **5**: myricitrin **8** 0.5 μM, **6**: myricitrin **8** 10 μM, **7**: metformin 10 μM, **8**: (−)-epicatechin **6** 0.5 μM, **9**: (−)-epicatechin **6** 10 μM, **10**: *D*-(+)-pinitol **5a** 0.5 μM, and **11** and **12**: *D*-(+)-pinitol **5a** 10 μM); Figure S3a–c. Original Western blot images of membrane 3 for the immunoblot analysis (lane **1**: vehicle control, **2**: metformin 10 μM, **3**: naringenin **1** 0.5 μM, **4**: naringenin **1** 10 μM, **5**: quercitrin **8** 0.5 μM, **6**: quercitrin **8** 10 μM, **7**: (−)-pinitol **5b** 0.5 μM, **8**: (−)-pinitol **5b** 10 μM, **9**: (−)-epicatechin **6** 0.5 μM, **10**: (−)-epicatechin **6** 10 μM, and **11:** *D*-(+)-pinitol **5a** 0.5 μM, and **12**: *D*-(+)-pinitol **5a** 10 μM).

## Sample Availability

Samples of the compounds are not available from the authors.
