# Supplementary material for: Antihyperglycemic Properties of Extracts and Isolated Compounds from Australian Acacia saligna on 3T3-L1 Adipocytes
Source: Molecules. 2023 May 12;28(10):4054. doi: 10.3390/molecules28104054 (PMC10223216; doi:10.3390/molecules28104054)
Supplement: Supplementary file 1 [file molecules-28-04054-s001.zip › molecules-2371093-supplementary.pdf]

Supplementary Information

Antihyperglycemic properties of extracts and isolated compounds from Australian *Acacia saligna* on 3T3-L1 adipocytes

Anjar P. Asmara [1], Anchalee Prasansuklab [2,3], Anchalee Chiabchalard[2,4], Hui Chen[5] and Alison T. Ung [1,*]

[1] School of Mathematical and Physical Sciences, Faculty of Science, University of Technology Sydney, Ultimo, NSW 2007, Australia
[2] Natural Products for Neuroprotection and Anti-Ageing Research Unit, Chulalongkorn University, Bangkok 10330, Thailand
[3] College of Public Health Sciences, Chulalongkorn University, Bangkok, 10330, Thailand
[4] Department of Clinical Chemistry, Faculty of Allied Health Sciences, Chulalongkorn University, Bangkok 10330, Thailand
[5] School of Life Sciences, Faculty of Science, University of Technology Sydney, Ultimo, NSW 2007, Australia
* Correspondence: alison.ung@uts.edu.au

Table of contents **Page**

Table S1. The extraction result from dried (250g) flowers, leaves, or barks of *A. saligna*. 2

Table S2. Viable 3T3-L1 adipocytes treated with isolates for 24, 48, and 72 h 3

Table S3. The estimated ROS level of adipocytes exposed to isolated compounds for 48 h 4

Table S4. Observed data of glucose uptake simulation with the fluoroprobe 2-NBDG assay for all extracts on the 3T3-L1 adipocytes 4

Table S5. Observed data of glucose uptake simulation with 2-NBDG fluorescence assay for isolated compounds on the 3T3-L1 adipocytes 5

Table S6. Quantitative data of the ratio of expressed p-AMPK-α to AMPK-α (%) by adipocytes exposed to the tested MeOH extracts 5

Table S7. Quantitative data of the ratio of expressed p-AMPK-α to AMPK-α (%) by adipocytes exposed to the tested isolates 6

Figure S1a-c. Original Western blot images of membrane 1 for the immunoblot analysis (lane **1**: vehicle control, **2**: metformin 10 μM, **3**: FL-MeOH 12.5 μg/mL, **4**: FL-MeOH 50 μg/mL, **5**: LF-MeOH 12.5 μg/mL, **6**: LF-MeOH 50 μg/mL, **7**: BK-MeOH 12.5 μg/mL, **8**: BK-MeOH 50 μg/mL, **9**: compound **2** 0.5 μM, **10**: compound **2** 10 μM, **11**: isosalipurposide **3** 0.5 μM, and **12**: isosalipurposide **3** 10 μM) 7-9

Figure S2a-c. Original Western blot images of membrane 2 for the immunoblot analysis (lane **1**: vehicle control, **2**: metformin 10 μM, **3**: quercitrin **4** 0.5 μM, **4**: quercitrin **4** 10 μM, **5**: myricitrin **8** 0.5 μM, **6**: myricitrin **8** 10 μM, **7**: metformin 10 μM, **8**: (−)-epicatechin **6** 0.5 μM, **9**: (−)-epicatechin **6** 10 μM, **10**: *D*-(+)-pinitol **5a** 0.5 μM, and **11** and **12**: *D*-(+)-pinitol **5a** 10 μM) 10-12

Figure S3a-c. Original Western blot images of membrane 3 for the immunoblot analysis (lane **1**: vehicle control, **2**: metformin 10 μM, **3**: naringenin **1** 0.5 μM, **4**: naringenin **1** 10 μM, **5**: quercitrin **8** 0.5 μM, **6**: quercitrin **8** 10 μM, **7**: (−)-pinitol **5b** 0.5 μM, **8**: (−)-pinitol **5b** 10 μM, **9**: (−)-epicatechin **6** 0.5 μM, **10**: (−)-epicatechin **6** 10 μM, and **11:** *D*-(+)-pinitol **5a** 0.5 μM, and **12**: *D*-(+)-pinitol **5a** 10 μM) 13-15

Table S1. The extraction results from dried (250g) flowers, leaves, or bark of *A. saligna*.

Type of Extract	ID, Mass of Extract (g)		
	**Dried Flowers (FL)**	**Dried Leaves (LF)**	**Dried Barks (BK)**
Hexane (hex)	FL-hex, 1.71	LF-hex, 3.08	BK-hex, 0.68
Dichloromethane (DCM)	FL-DCM, 1.79	LF-DCM, 4.98	BK-DCM, 2.12
Methanol (MeOH)	FL-MeOH, 26.16	LF-MeOH, 25.37	BK-MeOH, 18.26
Water (H_2O)	FL-H_2O, 36.31	LF-H_2O, 13.32	BK-H_2O, 4.34

Table S2. Viable 3T3-L1 adipocytes treated with isolates for 24, 48, and 72 h.

Sample	Incubation (h)	Viable adipocytes (%) at concentrations of (μM)			
		15.63	31.25	62.5	125
Vehicle (treatment-free)	24	100 ± 1.81			
	48	100 ± 2.52			
	72	100 ± 1.36			
Naringenin 1	24	98.67 ± 3.21	99.29 ± 3.58	97.92 ± 3.91	93.54 ± 1.92
	48	91.82 ± 8.36	98.59 ± 4.79	98.18 ± 4.63	94.33 ± 3.55
	72	92.65 ± 5.58	84.54 ± 6.28	87.11 ± 7.49	78.31 ± 2.28**
Naringenin-7O-*α*-L-arabinopyranose 2	24	100.6 ± 1.39	97.51 ± 1.27	97.53 ± 2.69	96.47 ± 3.10
	48	96.53 ± 8.24	94.4 ± 6.88	91.69 ± 6.60	90.01 ± 10.17
	72	78.09 ± 4.08*	78.99 ± 5.77*	89.79 ± 5.81	90.06 ± 4.65
Isosalipurposide 3	24	93.75 ± 0.57*	96.26 ± 1.82	96.65 ± 2.25	96.92 ± 1.87
	48	93.21 ± 0.10	93.78 ± 2.31	95.64 ± 3.49	95.71 ± 1.44
	72	100.2 ± 4.14	101 ± 3.92	101 ± 4.02	101.3 ± 3.07
Quercitrin 4	24	97.81 ± 1.83	99.16 ± 1.23	99.1 ± 0.98	98.46 ± 1.56
	48	95.74 ± 1.42	96.33 ± 0.94	98.02 ± 1.59	96.99 ± 2.34
	72	98.54 ± 1.3	98.76 ± 1.56	99.32 ± 1.59	99.92 ± 2.12
D-(+)-pinitol 5a	24	93.58 ± 3.19	92.6 ± 2.69	93.65 ± 3.39	95.3 ± 3.63
	48	94.42 ± 4.16	97.64 ± 3.42	95.06 ± 3.84	95.32 ± 4.72
	72	97.92 ± 4.66	98.62 ± 4.07	98.19 ± 3.04	97.54 ± 3.11
(–)-Pinitol 5b	24	91.36 ± 2.37	91.89 ± 2.19	91.36 ± 1.73	93.41 ± 2.65
	48	92.01 ± 3.03	93.7 ± 3.96	93.45 ± 3.92	93.35 ± 3.85
	72	94.38 ± 2.90	93.97 ± 4.30	93.58 ± 3.58	95.94 ± 3.92
(–)-Epicatechin 6	24	98.17 ± 1.55	100.1 ± 0.94	100 ± 0.41	99.31 ± 0.77
	48	97.94 ± 3.33	97.78 ± 4.22	98.39 ± 4.32	97.73 ± 1.01
	72	88.74 ± 7.24	94.62 ± 2.01	92.28 ± 1.38	86.87 ± 6.90*
2,4-Di-*t*-butylphenol 7	24	100.7 ± 1.05	98.31 ± 2.16	101.2 ± 1.52	101.5 ± 1.60
	48	97.8 ± 4.18	90.83 ± 1.07	101.4 ± 3.42	101.5 ± 4.69
	72	98.8 ± 1.35	82 ± 5.34*	88.6 ± 10.96	90.2 ± 7.58
Myricitrin 8	24	98.77 ± 0.29	97.99 ± 3.07	100 ± 0.70	100.4 ± 1.57
	48	95.91 ± 2.47	94.4 ± 3.99*	95.05 ± 3.17	95.6 ± 1.52
	72	104.6 ± 5.07	105.4 ± 5.27	102.7 ± 2.84	104 ± 3.47
3-Hydroxy-5-(2-aminoethyl) dihydrofuran-2(3*H*)-one 9	24	100.2 ± 1.41	100.9 ± 1.80	97.23 ± 4.11	98.67 ± 1.84
	48	96.19 ± 5.80	96.76 ± 5.56	95.56 ± 6.77	96.84 ± 3.16
	72	82.7 ± 3.24*	95.72 ± 3.18	89.75 ± 6.28	85.22 ± 4.60*

* $p = 0.01$; ** $p = 0.003$, *p* values were from indicated samples *vs* vehicle control ($n = 3$, one-way ANOVA, with Dunnett post hoc tests).

Table S3. The estimated ROS level of adipocytes exposed to isolated compounds for 48 h.

Sample	Cellular ROS level (%) at the corresponding concentration (μM)	
	**0.5**	**10**
**Vehicle**	100 ± 1.87	
**Naringenin 1**	98.7 ± 2.89	75.82 ± 6.20*
**Naringenin-7-O-*α*-*L*-arabinopyranoside 2**	99.06 ± 10.95	76.64 ± 5.16
**Isosalipurposide 3**	98.33 ± 2.27	80.13 ± 7.52
**2,4-Di-t-buylphenol 7**	99.95 ± 0.93	87.94 ± 5.29
**Quercitrin 4**	102.9 ± 0.36	87.65 ± 0.72
**Myricitrin 8**	100.5 ± 7.66	78.64 ± 6.14
**3-Hydroxy-5-(2-aminoethyl) dihydrofuran-2(3H)-one 9**	99.6 ± 6.24	92.67 ± 3.20
**(–)-Pinitol 5b**	88.76 ± 2.96	79.57 ± 6.40
**(–)-Epicatechin 6**	105.5 ± 3.99	71.45 ± 4.82**
***D*-(+)-pinitol 5a**	89.84 ± 0.88	69.24 ± 3.90**
**NAC 5 mM**	78.28 ± 2.83	
**NAC 10 mM**	64.74 ± 2.24***	
**Undifferentiated cells**	53.79 ± 5.41****	

$p = 0.05$, ** $p = 0.002$, *** $p = 0.0003$, and **** $p = 0.000003$ were from the ROS level of the indicated samples *vs* vehicle control ($n = 3$, one-way ANOVA, with Tukey post hoc tests).

Table S4. Observed data of glucose uptake simulation with the fluoroprobe 2-NBDG assay for methanolic extracts on the 3T3-L1 adipocytes.

Sample	2-NBDG uptake percentage (%)	
	**12.5 μg/mL**	**50 μg/mL**
**Vehicle**	100 ± 6.54	
**FL-MeOH**	141.5 ± 27.94	185.3 ± 41.52 **
**LF-MeOH**	113.5 ± 6.1	198 ± 42.61 **
**BK-MeOH**	118.3 ± 9.517	161.6 ± 10.76
**Insulin 100 nM**	140.6 ± 18.36	
**Metformin 10 μM**	138 ± 28.26	

** $p = 0.007$ for FL-MeOH and ** $p = 0.006$ for LF-MeOH compared to the vehicle control ($n = 3$, one-way ANOVA, with Dunnett post hoc tests).

Table S5. Observed data of glucose uptake simulation with 2-NBDG fluorescence assay for isolated compounds on the 3T3-L1 adipocytes.

Sample	2-NBDG uptake percentage (%)	
	0.5 µM	10 µM
**Vehicle**	100 ± 6.54	
**Naringenin 1**	89.3 ± 9.47	127.3 ± 15
**Naringenin-7-O-*α*-*L*-arabinopyranoside 2**	107.6 ± 7.89	156.4 ± 22.26
**Isosalipurposide 3**	110.7 ± 13.26	161 ± 39.47
**Quercitrin 4**	101.6 ± 14.07	151 ± 10.03
***D*-(+)-pinitol 5a**	108.5 ± 11.36	143.9 ± 12.56
**(−)-Pinitol 5b**	96.99 ± 3.25	125.6 ± 13.27
**(−)-Epicatechin 6**	108.3 ± 1.12	187.9 ± 41.95*
**2,4-Di-*t*-buylphenol 7**	86.39 ± 10.81	131.2 ± 21.57
**Myricitrin 8**	122.7 ± 10.74	152.3 ± 24.02
**3-Hydroxy-5-(2-aminoethyl) dihydrofuran-2(3H)-one 9**	89.56 ± 7.20	96.64 ± 10.97
**Insulin 100 nM**	140.6 ± 18.36	
**Metformin 10 µM**	138 ± 28.26	

**p* = 0.01, *p* value was from the indicated sample against the vehicle control (*n* = 3, one-way ANOVA, with Dunnett post hoc tests).

Table S6. Quantitative data of the ratio of expressed p-AMPK-*α* to AMPK-*α* (%) by adipocytes exposed to methanolic extracts.

Treatment	Concentration	Ratio of p-AMPK-*α* to AMPK-*α* (%)
**Vehicle (treatment-free)**	-	100 ± 17.16
**Metformin**	10 µM	166.4 ± 14.08
**FL-MeOH**	12.5 µg/mL	128.1 ± 10.56
	50 µg/mL	177 ± 16.98*
**LF-MeOH**	12.5 µg/mL	128.9 ± 12.97
	50 µg/mL	158.5 ± 13.76
**BK-MeOH**	12.5 µg/mL	129.7 ± 22.79
	50 µg/mL	149.1 ± 25.85

* *p* = 0.02, *p* value was of the sample against the vehicle control (*n* = 3, one-way ANOVA, with Tukey post hoc tests).

Table S7. Quantitative data of the ratio of expressed p-AMPK-α to AMPK-α (%) by adipocytes exposed to isolated compounds.

Sample	Concentration (μM)	Ratio of p-AMPK-α to AMPK-α (%)
**Vehicle**	-	100 ± 13.36
**Metformin**	10	191.8 ± 21.86**
**Naringenin 1**	0.5	123.8 ± 2.34
	10	148.4 ± 13.56
**Naringenin-7-O-α-*L*-arabinopyranoside 2**	0.5	139.1 ± 13.04
	10	211.8 ± 30.27***
**Isosalipurposide 3**	0.5	129.8 ± 14.7
	10	196.6 ± 20.33**
**Quercitrin 4**	0.5	110.7 ± 11.33
	10	148.6 ± 12.2
***D*-(+)-pinitol 5a**	0.5	91.72 ± 6.07
	10	98.61 ± 8.55
**(−)-Pinitol 5b**	0.5	94.05 ± 7.39
	10	102.7 ± 22.67
**(−)-Epicatechin 6**	0.5	99.62 ± 3.88
	10	143.2 ± 17.25
**Myricitrin 8**	0.5	109.4 ± 10.11
	10	156 ± 8.11

** $p = 0.003$, *** $p = 0.0002$, p values were of samples against the vehicle control ($n = 3$, one-way ANOVA, with Tukey post hoc tests).

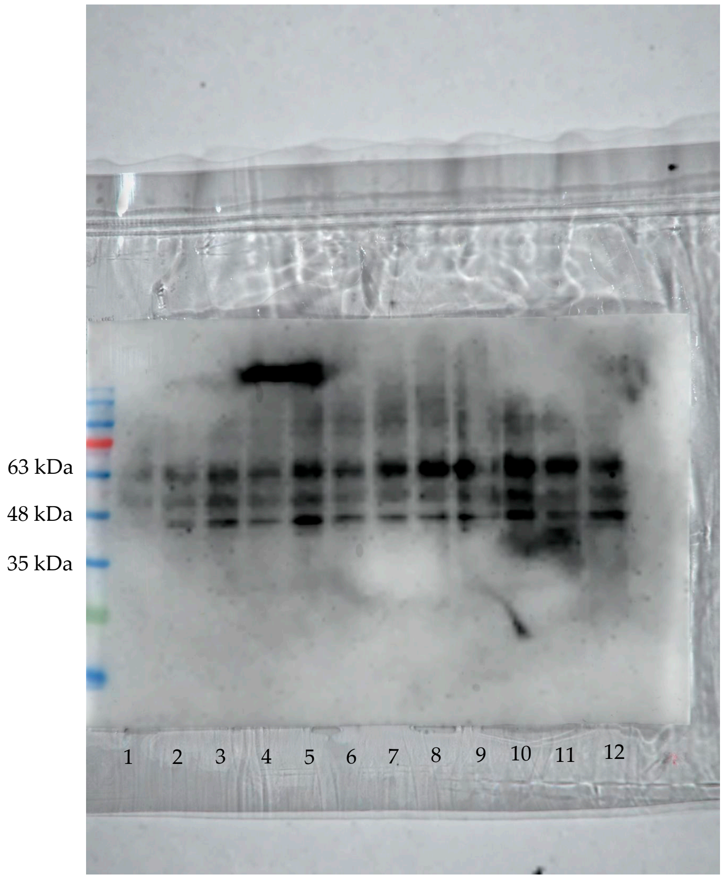

<u>Legend</u>
1: vehicle control
2: metformin 10 μM
3: FL-MeOH 12.5 μg/mL
4: FL-MeOH 50 μg/mL
5: LF-MeOH 12.5 μg/mL
6: LF-MeOH 50 μg/mL
7: BK-MeOH 12.5 μg/mL
8: BK-MeOH 50 μg/mL
9: compound **2** 0.5 μM
10: compound **2** 10 μM
11: isosalipurposide **3** 0.5 μM
12: isosalipurposide **3** 10 μM

(a) p-AMPK-α

Figure S1a. Original Western blot images of membrane 1 for the immunoblot analysis of p-AMPK.

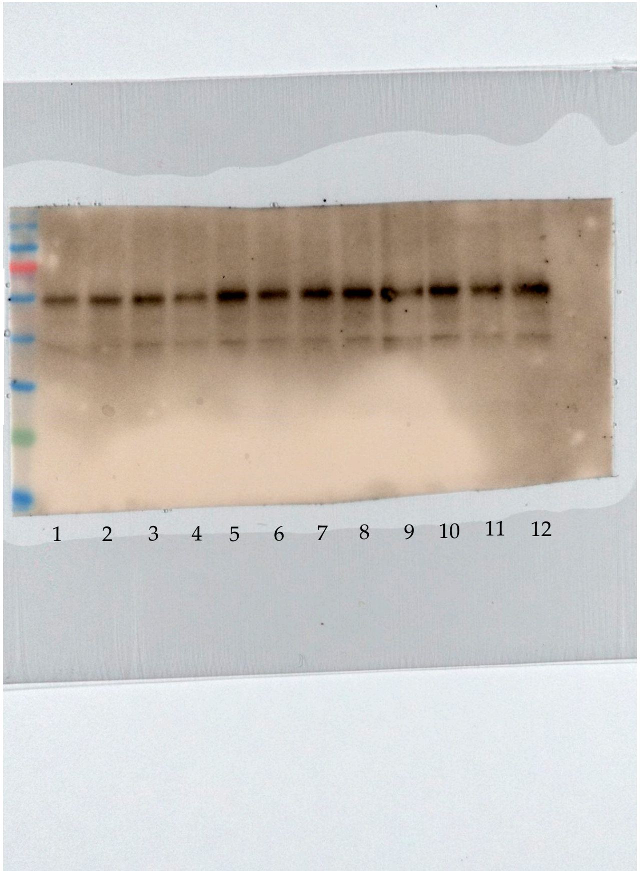

Legend
1: vehicle control
2: metformin 10 μM
3: FL-MeOH 12.5 μg/mL
4: FL-MeOH 50 μg/mL
5: LF-MeOH 12.5 μg/mL
6: LF-MeOH 50 μg/mL
7: BK-MeOH 12.5 μg/mL
8: BK-MeOH 50 μg/mL
9: compound **2** 0.5 μM
10: compound **2** 10 μM
11: isosalipurposide **3** 0.5 μM
12: isosalipurposide **3** 10 μM

(b) AMPK-α

Figure S1b. Original Western blot images of membrane 1 for the immunoblot analysis of AMPK.

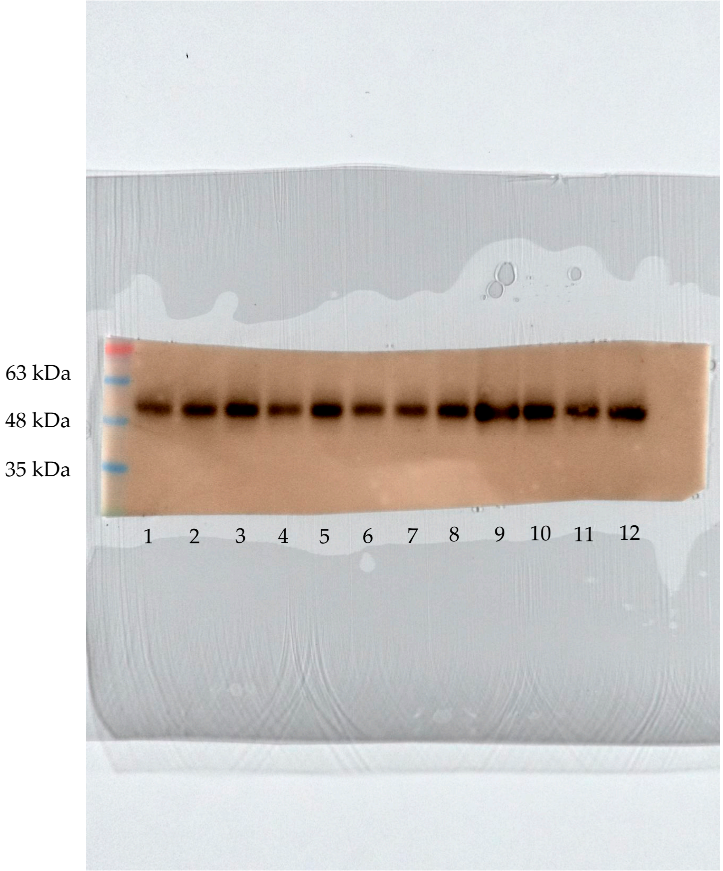

Legend
1: vehicle control
2: metformin 10 μM
3: FL-MeOH 12.5 μg/mL
4: FL-MeOH 50 μg/mL
5: LF-MeOH 12.5 μg/mL
6: LF-MeOH 50 μg/mL
7: BK-MeOH 12.5 μg/mL
8: BK-MeOH 50 μg/mL
9: compound **2** 0.5 μM
10: compound **2** 10 μM
11: isosalipurposide **3** 0.5 μM
12: isosalipurposide **3** 10 μM

(c) α-tubulin

Figure S1c. Original Western blot images of membrane 1 for the immunoblot analysis of α-tubulin

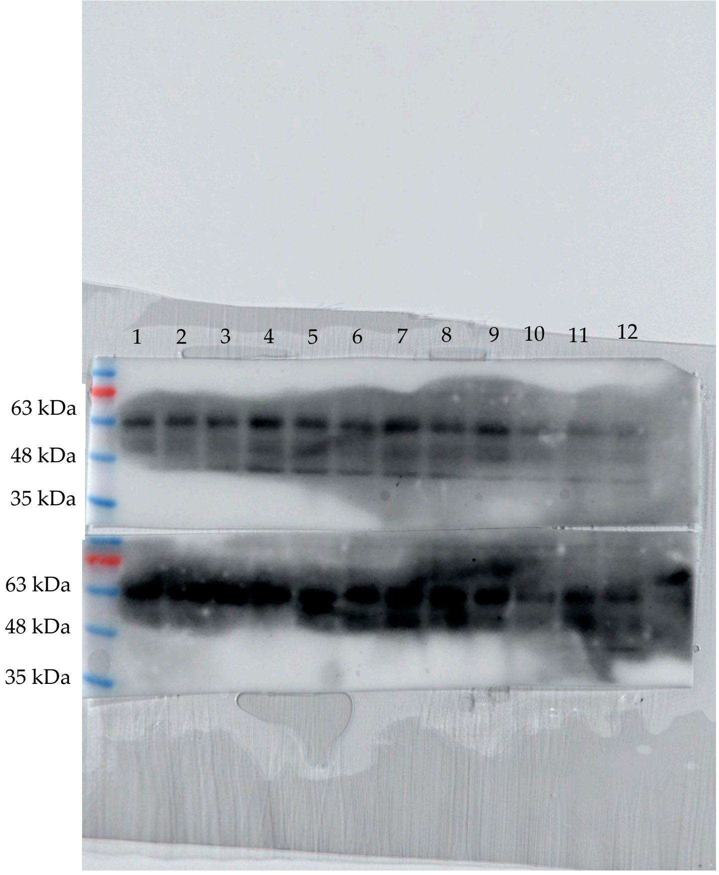

<u>Legend</u>
1: vehicle
2: metformin 10 μM
3: quercitrin **4** 0.5 μM
4: quercitrin **4** 10 μM
5: myricitrin **8** 0.5 μM
6: myricitrin **8** 10 μM
7: metformin 10 μM
8: (−)-epicatechin **6** 0.5 μM
9: (−)-epicatechin **6** 10 μM
10: *D*-(+)-pinitol **5a** 0.5 μM
11: *D*-(+)-pinitol **5a** 10 μM
12: *D*-(+)-pinitol **5a** 10 μM

(a) p-AMPK-α

Figure S2a. Original Western blot images of membrane 2 for the immunoblot analysis of p-AMPK

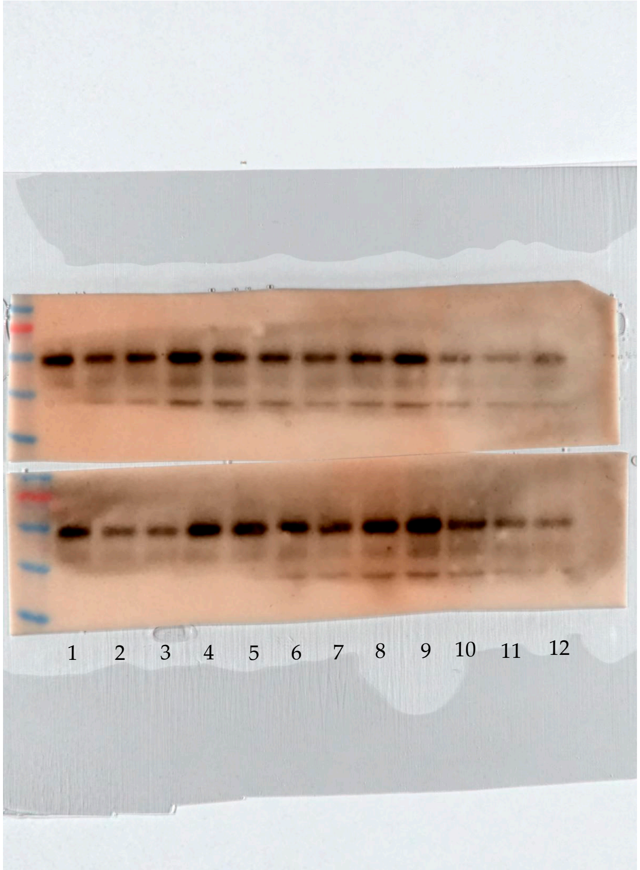

Legend
1: vehicle
2: metformin 10 μM
3: quercitrin **4** 0.5 μM
4: quercitrin **4** 10 μM
5: myricitrin **8** 0.5 μM
6: myricitrin **8** 10 μM
7: metformin 10 μM
8: (−)-epicatechin **6** 0.5 μM
9: (−)-epicatechin **6** 10 μM
10: *D*-(+)-pinitol **5a** 0.5 μM
11: *D*-(+)-pinitol **5a** 10 μM
12: *D*-(+)-pinitol **5a** 10 μM

(b) AMPK-α

Figure S2b. Original Western blot images of membrane 2 for the immunoblot analysis of AMPK

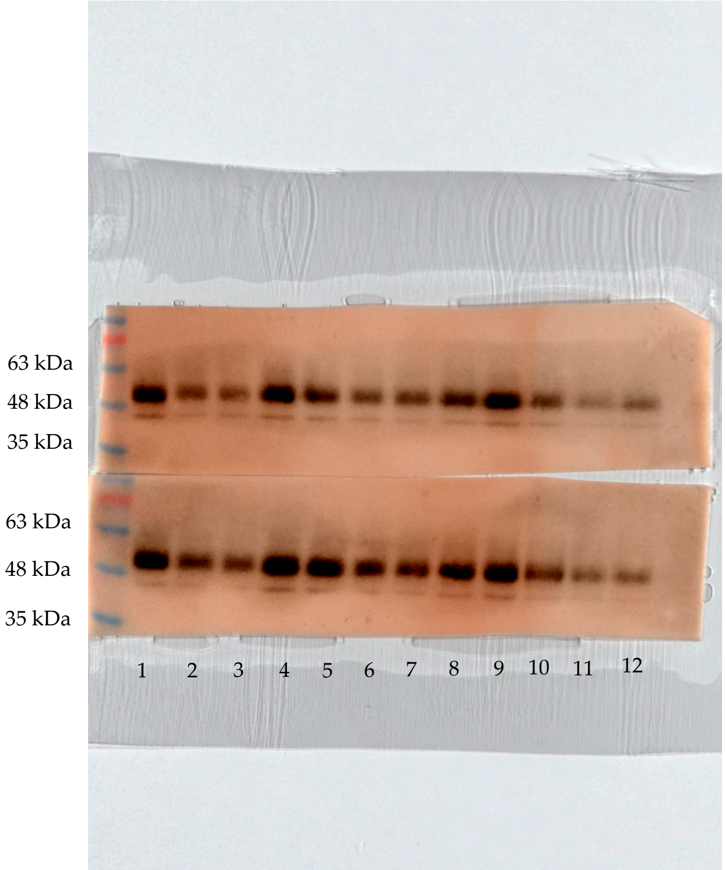

Legend

1: vehicle
2: metformin 10 μM
3: quercitrin **4** 0.5 μM
4: quercitrin **4** 10 μM
5: myricitrin **8** 0.5 μM
6: myricitrin **8** 10 μM
7: metformin 10 μM
8: (−)-epicatechin **6** 0.5 μM
9: (−)-epicatechin **6** 10 μM
10: *D*-(+)-pinitol **5a** 0.5 μM
11: *D*-(+)-pinitol **5a** 10 μM
12: *D*-(+)-pinitol **5a** 10 μM

(c) α-tubulin

Figure S2c. Original Western blot images of membrane 2 for the immunoblot analysis of α-tubulin

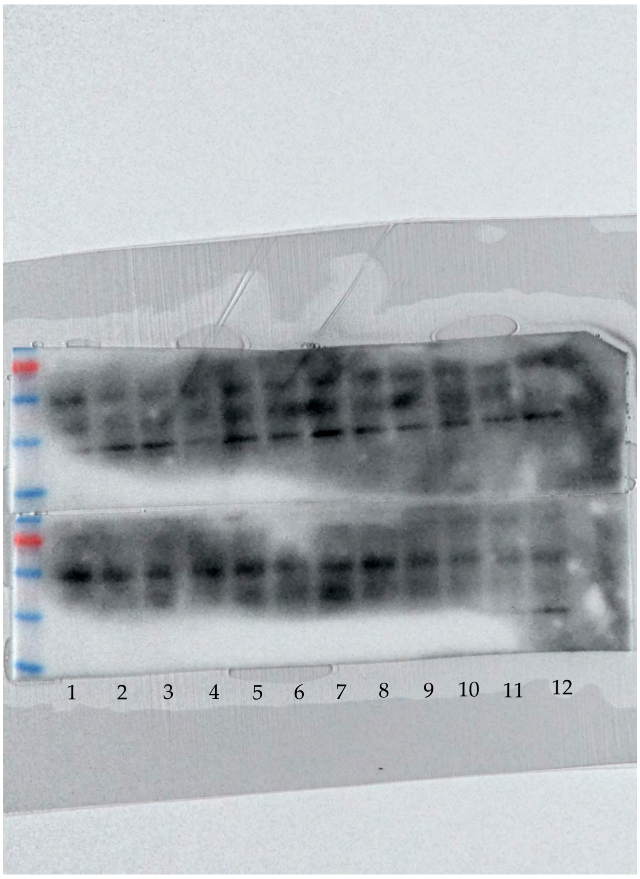

Legend

1: vehicle
2: metformin 10 μM
3: naringenin **1** 0.5 μM
4: naringenin **1** 10 μM
5: quercitrin **4** 0.5 μM
6: quercitrin **4** 10 μM
7: (−)-pinitol **5b** 0.5 μM
8: (−)-pinitol **5b** 10 μM
9: (−)-epicatechin **6** 0.5 μM
10: (−)-epicatechin **6** 10 μM
11: *D*-(+)-pinitol **5a** 0.5 μM
12: *D*-(+)-pinitol **5a** 10 μM

(a) p-AMPK-α

Figure S3a. Original Western blot images of membrane 3 for the immunoblot analysis of p-AMPK

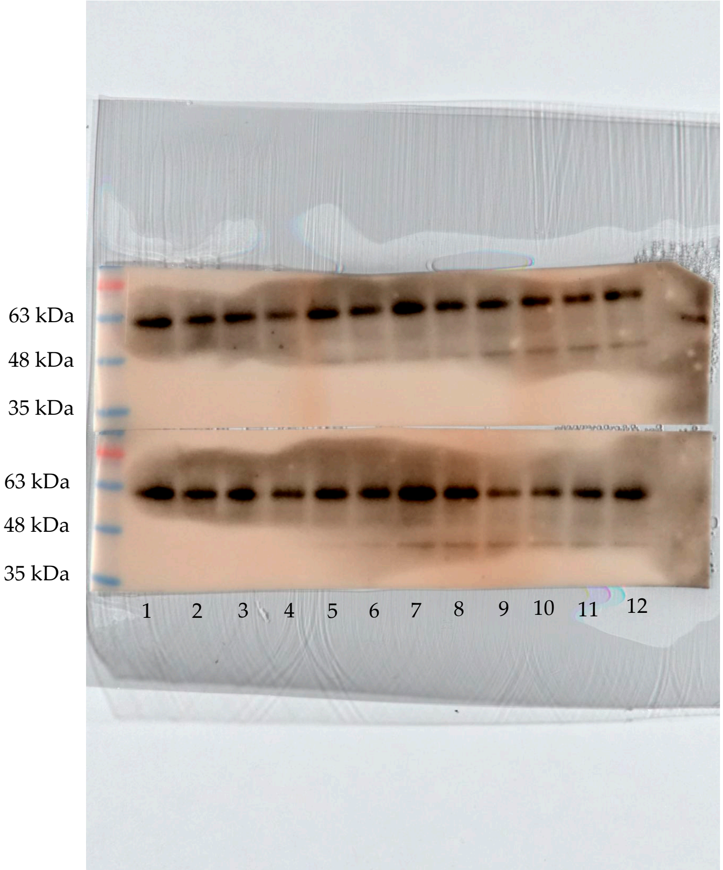

Legend

1: vehicle
2: metformin 10 μM
3: naringenin **1** 0.5 μM
4: naringenin **1** 10 μM
5: quercitrin **4** 0.5 μM
6: quercitrin **4** 10 μM
7: (−)-pinitol **5b** 0.5 μM
8: (−)-pinitol **5b** 10 μM
9: (−)-epicatechin **6** 0.5 μM
10: (−)-epicatechin **6** 10 μM
11: *D*-(+)-pinitol **5a** 0.5 μM
12: *D*-(+)-pinitol **5a** 10 μM

(b) AMPK-α

Figure S3b. Original Western blot images of membrane 3 for the immunoblot analysis of AMPK

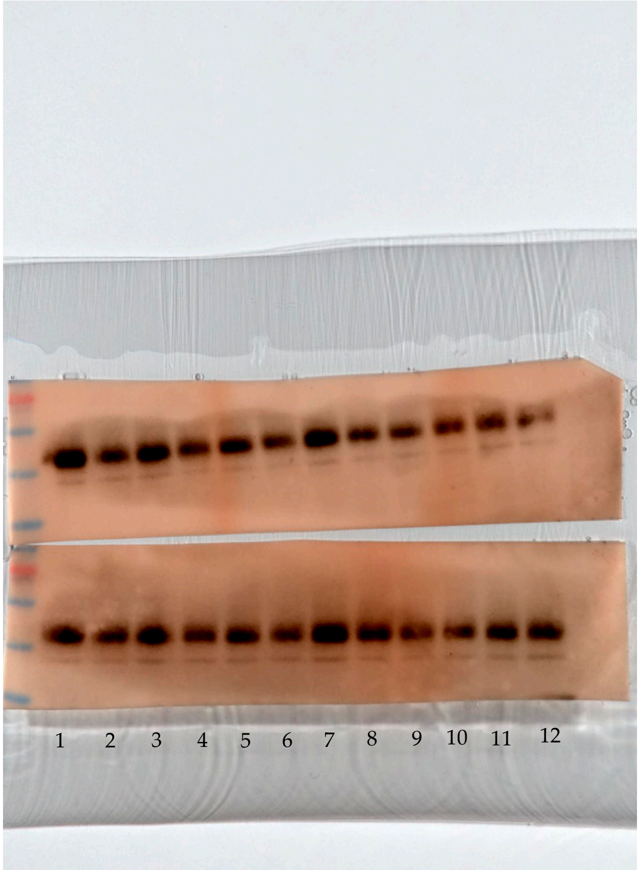

<u>Legend</u>
1: vehicle
2: metformin 10 μM
3: naringenin **1** 0.5 μM
4: naringenin **1** 10 μM
5: quercitrin **4** 0.5 μM
6: quercitrin **4** 10 μM
7: (−)-pinitol **5b** 0.5 μM
8: (−)-pinitol **5b** 10 μM
9: (−)-epicatechin **6** 0.5 μM
10: (−)-epicatechin **6** 10 μM
11: *D*-(+)-pinitol **5a** 0.5 μM
12: *D*-(+)-pinitol **5a** 10 μM

(c) α-tubulin

Figure S3c. Original Western blot images of membrane 3 for the immunoblot analysis of α-tubulin
